# Competing Interactions of RNA-Binding Proteins, MicroRNAs, and Their Targets Control Neuronal Development and Function

**DOI:** 10.3390/biom5042903

**Published:** 2015-10-23

**Authors:** Amy S. Gardiner, Jeffery L. Twiss, Nora I. Perrone-Bizzozero

**Affiliations:** 1 Department of Neurosciences, University of New Mexico Health Sciences Center, Albuquerque, NM 87131, USA; E-Mail: agardiner@salud.unm.edu; 2 Department of Biological Sciences, University of South Carolina, Columbia, SC 29208, USA; E-Mail: TWISS@mailbox.sc.edu; 3 Department of Psychiatry and Behavioral Sciences, University of New Mexico Health Sciences Center, Albuquerque, NM 87131, USA

**Keywords:** RNA-binding proteins, HuD, KSRP, ZBP1 (IMP1), target transcripts, neurons

## Abstract

Post-transcriptional mechanisms play critical roles in the control of gene expression during neuronal development and maturation as they allow for faster responses to environmental cues and provide spatially-restricted compartments for local control of protein expression. These mechanisms depend on the interaction of *cis*-acting elements present in the mRNA sequence and trans-acting factors, such as RNA-binding proteins (RBPs) and microRNAs (miRNAs) that bind to those *cis*-elements and regulate mRNA stability, subcellular localization, and translation. Recent studies have uncovered an unexpected complexity in these interactions, where coding and non-coding RNAs, termed competing endogenous RNAs (ceRNAs), compete for binding to miRNAs. This competition can, thereby, control a larger number of miRNA target transcripts. However, competing RNA networks also extend to competition between target mRNAs for binding to limited amounts of RBPs. In this review, we present evidence that competitions between target mRNAs for binding to RBPs also occur in neurons, where they affect transcript stability and transport into axons and dendrites as well as translation. In addition, we illustrate the complexity of these mechanisms by demonstrating that RBPs and miRNAs also compete for target binding and regulation.

## 1. Introduction

### 1.1. Post-Transcriptional Regulation

Gene expression is a complex and tightly-regulated process. In addition to transcription, post-transcriptional mechanisms, such as mRNA processing, stability, transport, and local translation, are critical for controlling gene expression. Of interest to this review, mechanisms that influence mRNA stability are particularly important for neural genes, as a great number are regulated in this manner. Specific RNA-binding proteins (RBPs), both stabilizing and destabilizing factors, and to some degree microRNAs (miRNAs), which generally function as destabilizers, mediate the stability and, therefore, determine the half-life of a large number of mRNAs. The half-life of a particular mRNA depends on sequences in the transcript itself, generally located in the 3' UTR of the mRNA, as well as the RBPs that bind to these sequences [1,2,3]. Some mRNAs are unstable, with a very short half-life while others are quite stable with very long half-lives [4]. The most studied instability-conferring sequence is the Adenylate-Uridylate Rich Element (ARE) [5,6]. AREs were initially identified in oncogenes, immediate early genes, and cytokines, but they are now recognized across multiple gene families. In fact, a recent database of ARE-containing mRNAs (ARED) [7] lists over 1500 mRNAs. Since the ARED is limited to mRNAs containing canonical ARE sequences, this database represents only a fraction of the total number of unstable mRNAs. Nevertheless, this information has proven very useful as shown by a recent study demonstrating the post-transcriptional regulation of many of the mRNAs listed in ARED [8].

### 1.2. mRNA Stability in Neurons

About 20% of all neuronal mRNAs in the mouse forebrain contain ARE sequences in their 3' UTR or interact with ARE-binding proteins (ARE-BPs), such as HuD [9], suggesting that these transcripts may be subjected to similar post-transcriptional regulation. In fact, studies from our group and others have demonstrated that HuD stabilizes not only the mRNA for the neuronal GAP-43 protein but also many other neuronal transcripts (for review see [10]). Furthermore, besides GAP-43, many ARE-containing mRNAs listed in the ARED database are expressed in the nervous system, such as the dopamine D1 receptor, SNAP-25, and FMR1 [7,8]. Using RNA immunoprecipitation followed by DNA microarray (RIP-Chip) analyses, Keene’s group identified potential targets of HuB in neuronally-differentiated P19 cells including integrin β, cyclins A2, B1, C, D1 and D2, IGF-2, neurofilament-M, zif268, TGF β, N-cadherin, c-myc, and N-myc mRNAs [11]. In subsequent studies by the same group, other Hu proteins, HuR and HuD, were shown to interact with CaMKIIα, Neuritin, Homer 1, Actin, and Neuroligin 1 and 3 in an activity-dependent manner [12]. Likewise, using affinity chromatography, Malter and co-workers were able to isolate multiple HuR-bound mRNAs from hippocampal tissue post-seizure including APP, MBP, PLP, Bcl2, neurogranin, calmodulin, SV2a, doublecortin, PKC gamma, calcineurin, glutamate NR1 receptor, SCG-10, syntaxin, VAMP-1, and β-catenin [13]. Finally, using a combination of RIP and recombinant protein pull-downs, we identified a large number of HuD target transcripts from mouse forebrain and characterized the recognition motifs and biological pathways enriched with these transcripts [9]. Consistent with the known functions of HuD, gene ontology analyses revealed that its targets are enriched in signaling pathways involved in neuronal differentiation and that many of these mRNAs encode other RBPs, translation factors, and actin-binding proteins. Altogether, these studies suggest that post-transcriptional regulation by changes in mRNA stability may be more widespread in the nervous system than previously thought.

### 1.3. Mechanisms Controlling mRNA Stability

In mammalian cells, deadenylation is the first step in the degradation of mRNAs containing ARE sequences followed by either 3'–5' exonucleolytic cleavage or decapping and 5' to 3' decay [14]. Like protein degradation, mRNA degradation is a complex process involving several steps and multiprotein complexes, such as the exosome, which is responsible for the 3' to 5' degradation pathway [15,16,17,18]. Alternatively, ARE-containing mRNAs can be degraded via decapping and 5'–3' exonuclease digestion [19,20]. Some RBPs, such as Hu proteins, increase the stability of mRNAs they interact with, whereas others such as TTP, AUF1, and KSRP, act as destabilizing factors [21,22,23]. Furthermore, it is now clear that mRNA degradation is not a default pathway but is regulated by specific extracellular signals [8,24,25,26,27].

Genes regulated by mRNA stability belong to different functional families, from transcription factors and structural proteins to membrane receptors and signaling molecules. After transcription, there are many possible fates for each mRNA and stabilization of an mRNA can synergistically increase the amount of the transcript available for translation. Efficiency of translation can also be regulated to fine tune the amount of protein derived from an mRNA. The combination of transcriptional and posttranscriptional regulation enables not only faster responses but also more flexibility in the control of gene expression in different neuronal populations.

In this review, we will focus on a few RNA-binding proteins that are key players during neuronal development and describe their competitive interactions with each other, their mRNA targets, and miRNAs.

## 2. RNA-Binding Proteins

### 2.1. HuD and Other Members of ELAV-Like/Hu Protein Family

Hu proteins comprise a family of RBPs that were first identified as the targets of autoantibodies found in patients with paraneoplastic encephalomyelitis [28]. These proteins are homologs of embryonic lethal abnormal vision (ELAV), a *Drosophila* RNA-binding protein whose deletion was found to be lethal for flies [29]. There is one ELAV protein in *Drosophila*, however, four mammalian ELAV-like Hu proteins have been identified, including HuR (also known as HuA), HuB (also known as Hel-N1), HuC, and HuD. Three of these proteins (HuB, HuC, and HuD) are developmentally regulated and expressed in neurons [30]. The fourth, HuR, is expressed in multiple tissues. At the molecular level, all four ELAV-like Hu proteins contain three RNA recognition motifs (RRMs), a highly-conserved 80 amino acid region that was first recognized in splicing factors and poly(A)-binding proteins [31]. Hu proteins bind preferentially to AREs found in the 3'UTRs of many mRNAs involved in cell growth and differentiation [21,32].

In higher vertebrates, Hu proteins are expressed in early development and represent one of the earliest markers expressed in neurons [33]. HuR is the first protein to be expressed in chicken embryos where it is thought to be involved in cell proliferation [34]. HuR is mainly nuclear and shuttles to the cytosol [35,36]. HuC is present in both nuclear and cytosolic fraction while HuB and HuD are primarily cytosolic [37,38,39,40,41]. The expression of HuD coincides with the earliest stages of neuronal differentiation and is maintained through the maturation of neurons [34]. Similar types of expression patterns have been observed in the developing mouse [30] and rat [42]. The involvement of Hu proteins in different stages of neuronal differentiation was confirmed by overexpression and knock-out studies. Overexpression of HuB, HuC or HuD in PC12 cells and *in vivo* resulted in an increased rate of neuronal differentiation [38,41,43,44,45]. Down-regulation of these proteins in neural cell lines resulted in the opposite phenotype, with cells failing to grow neurites [44,46]. Likewise, HuD KO mice showed increased proliferation of neural precursors but decreased neuronal differentiation and decreased dendritic complexity [47,48]. HuC is also important for proper neuronal maturation as the KO mice were found to have increased brain glutamate levels and exhibit spontaneous seizures [49].

Although Hu proteins were initially described as early markers of development in certain mature neurons, significant levels of Hu proteins persist throughout life, particularly in the cortex and hippocampus. As shown in Bolognani *et al.* (2004), HuD protein is present in the soma and dendrites of pyramidal cells in the hippocampus and neocortex in close association with polysomes [39]. In contrast, HuD protein is not detected in the mature granule cells [40]. The spatial pattern of HuB expression is similar to that of HuD but distinct from HuC, which is normally expressed at high levels in dentate granule cells [30]. The function of these proteins in mature neurons is not well understood. The findings that HuD protein expression is increased during learning and memory [39,50,51] and after epileptic activity [12,52] suggest that these proteins may also play an important role in mechanisms of synaptic plasticity. Furthermore, genetic manipulations of HuD protein levels in adult mice result in similar learning and memory deficits as demonstrated by cognitive impairment in HuD overexpressing (HuD OE) or KO mice [48,53]. Altogether, these results support the notion that Hu proteins play a critical role in nervous system development and emphasize that HuD levels need to be tightly regulated for proper brain function.

Recently, two reports have suggested that HuD binding to 3' UTR sequences promotes the translation of mRNAs by interacting with cap-binding eIF4A proteins and the poly (A) tail [54,55]. Consistent with the role of poly (A) tail length in mRNA stability, we have previously shown that HuD preferentially binds and stabilizes capped GAP-43 mRNAs with long poly (A) tails [56]. In addition, HuD may relieve miRNA-mediated repression, which promotes dissociation of eIF4A proteins, by restoring the interaction between the mRNA cap and the poly (A) tail [55]. HuD may also exert an additional function in the control of localized mRNAs, as transported RNA-protein complexes are translationally suppressed and HuD has also been linked to transport and localization of neuronal mRNAs. For example, the cis-element required for GAP-43 mRNA localization into PNS axons was mapped to the ARE/HuD binding site in its 3' UTR [57]. HuD has also been implicated in axonal localization of other neuronal mRNAs including Tau, Neuritin/CPG15, Kv.1.1 and CaMKIIα mRNAs [58,59,60,61]. Since the majority of these HuD targets are locally translated in response to specific signaling mechanisms such as those involving mTOR and PKC [60,61,62], it will be critical to determine how HuD’s function can switch from stabilizing and targeting bound mRNAs to facilitating their translation into proteins.

### 2.2. KSRP

KSRP (K homology Splicing Regulatory Protein) was originally identified as an RNA-binding protein that enhances retention of a neuronal-specific exon in the c-src mRNA [63]. This protein is also known as FBP-2, a member of the Far upstream Binding Protein (FBP) family identified for their binding to the c-myc enhancer. In addition to being localized in the nucleus, KSRP is present in the cytoplasm where it binds to several unstable ARE-containing mRNAs promoting their degradation via an exosome-mediated pathway [17,25,64]. KSRP phosphorylation by p38 decreases the binding of this RNA-binding protein to its targets, suggesting that mRNA degradation is not a default pathway but it is regulated by specific extracellular signals [24,25,26,27]. KSRP is expressed in the nervous system in both neurons and glia [65,66,67] and is a homolog of chicken ZBP-2, a protein that is required for localizing β-actin mRNA to growth cones [68] and rat MARTA-1, a protein that transports MAP-2 mRNA to dendrites [69]. Additionally, KSRP has been shown to bind to the survival motor neuron (SMN) protein and localize to motoneuron axons [70].

KSRP is best characterized as a destabilizing factor for mRNAs, as it recruits the exosome to induce their degradation [17,64]. This has been most extensively studied in the immune system, and more recently in astrocytes, where KSRP promotes the decay of interleukins, cytokines and inducible nitric oxide (iNOS) mRNAs [22,67]. Despite the fact that KSRP is expressed at high levels in neurons [71], the function of KSRP in these cells remains poorly understood. Our recently published work shows a direct role for KSRP in the control of neuronal mRNA stability and rate of axonal outgrowth [72].

In addition to promoting mRNA decay, KSRP has also been shown to promote the biogenesis of a subset of miRNAs, such as let-7a and miR-1 [73]. DNA damage induces the phosphorylation of KSRP by ATM, resulting in increased processing of particular miRNAs [74]. Furthermore, KSRP and hnRNPA1 act antagonistically in regulating the biogenesis of let-7a [75].

### 2.3. ZBP1/IMP1

In addition to ARE sequences, many mRNAs contain cis-elements in their 3' UTRs that confer subcellular localization through interaction with RBPs. The 54 nucleotide cis-element in β-actin’s 3' UTR that confers localization to dendrites and axons has been termed a “zipcode”. Zipcode binding protein 1 (ZBP1) was first identified as an mRNA transport protein binding to the chicken β-actin 3' UTR zipcode [76]. This protein is the orthologue of the human insulin-like growth factor 2 mRNA-binding protein 1 (IMP-1), which was identified as a RNA-binding protein associated with tau mRNA in axons [58]. Similar to the ELAV-like/Hu proteins, ZBP1 has been shown to bind to many other cellular mRNAs [77,78]. ZBP1 and its orthologues have been implicated in many aspects of RNA regulation, including intracellular RNA localization, stability, and translational control [79,80].

## 3. Competition between mRNAs for Binding to RBPs

**Figure 1:**
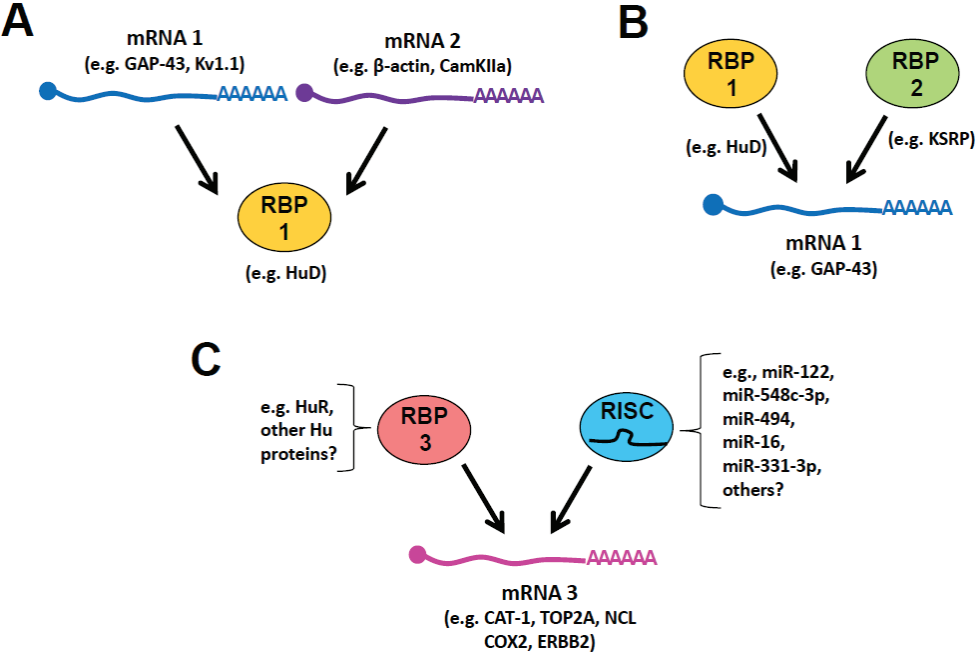
Examples of competing RBP target interactions: (**A**) mRNA1 and mRNA 2 compete for binding to RBP1; (**B**) RBP1 competes with RBP2 for binding to mRNA 1; and (**C**) RBP1 competes with the miRNA-associated RISC complex for binding to mRNA 3. Examples of the mRNAs, RBPs, and miRNAs associated with these three types of competitive interactions are indicated in each figure (see text for further details).

As mentioned above, a significant number of neural mRNAs contain AREs in their 3' UTRs, and they can be bound by various ARE-binding proteins. It is likely that many of these transcripts have similar temporal expression and localization patterns and may, therefore, compete for binding to the same protein. As shown in Figure 1A, two or more mRNAs can compete for the binding of the same RBP. For instance, GAP-43 and β-actin mRNAs compete for binding to ZBP1 that is expressed in limiting quantities in adult neurons [81]. Both transcripts localize into axons; overexpression of the localization element of either β-actin or GAP-43 mRNAs can prevent axonal localization of the other transcript, and increasing ZBP1 protein, rescues this deficit [57,81]. Interestingly, the axonally-synthesized β-actin and GAP-43 proteins generate distinct growth morphologies, with β-actin promoting axonal branching and GAP-43 inducing axonal elongation [82]. In DRG neurons, the GAP-43 gene is transcriptionally induced by axonal injury, but β-actin transcription is not significantly changed; this raises the interesting possibility that the increased levels of endogenous GAP-43 mRNA are able to displace β-actin transcripts from ZBP1, promoting axonal growth and regeneration [57,81]. As noted above, HuD has also been implicated in axonal localization of other neuronal mRNAs [58,59], suggesting that other mRNAs may compete for interaction with HuD in axons.

Another interesting example of target mRNA competition for binding to HuD is the interaction involving the competition of Kv1.1 and CamKIIα mRNAs for this RBP [60]. Expression of Kv1.1, an ion channel that is locally translated in neuronal processes, is controlled by miR-129 and HuD in a mTORC1 kinase-dependent manner. When mTORC1 is active, miR-129 represses Kv1.1 translation, and when mTORC1 is inhibited, HuD binds to and promotes Kv1.1 translation. HuD binding to Kv1.1 occurs as a result of the degradation of other high-affinity targets of HuD, such as CamKIIα, GAP-43, and Homer 1a, when mTOR is inactive. This was further demonstrated by the finding that overexpression of CamKIIα 3' UTR prevented HuD-mediated increases in Kv1.1 translation. Thus, in this instance, several mRNAs compete for binding of HuD, and HuD competes with miR-129 for binding to Kv1.1 mRNA [60].

## 4. Competition *vs.* Cooperation of RBPs

Neuronal ARE-BPs, such as HuD, ZBP1, and KSRP regulate many mRNAs and may compete with one or more proteins for the binding and regulation of their common targets (Figure 1B). There is some evidence for this competition, especially between proteins with antagonistic functions, such as those that affect RNA stability/instability. For example, KSRP’s binding to GAP-43 ARE serves to destabilize the mRNA resulting in its degradation [72]. As HuD stabilizes GAP-43 mRNA and promotes axonal outgrowth, we performed a competitive binding assay and showed that KSRP was able to displace HuD from the GAP-43 mRNA [71]. Overexpression of KSRP inhibited axonal outgrowth of hippocampal neurons, and this effect was reversed by overexpression of GAP-43 or a chimeric construct containing the GAP-43 coding region with a heterologous 3' UTR that confers axonal localization. When KSRP was knocked down or the binding sites for KSRP were removed, GAP-43 mRNA levels and axonal outgrowth were both increased [72].

There are also examples of cooperation among RBPs. ZBP1 and HuD bind together to the GAP-43 3' UTR in an RNA-dependent manner [57], suggesting that the binding sites of the proteins are non-overlapping. HuD and ZBP1 also cooperate for binding to tau mRNA [58]. This is different from the competitive binding of HuD and KSRP to GAP-43 mRNA [72] or the competition of HuD and ZBP1 for binding overlapping, yet distinct, domains in the β-actin mRNA zipcode [83]. Not surprisingly, the different modes of RBP interactions, competitive *vs.* cooperative, depend on whether these proteins bind overlapping *vs.* non-overlapping elements that allow both proteins to bind simultaneously to the target transcript such as in the case of HuD and ZPB1 for GAP-43 and tau.

## 5. Competition *vs.* Cooperation between RBPs and miRNAs

miRNAs are 22nt single-stranded RNAs that hybridize to complementary sequences primarily in the 3' UTR of mRNAs. This interaction most often serves to either prevent ribosome translation or destabilize the mRNA with the end result being a decrease in protein translation. A recent study indicates that the predominant effect of miRNA binding is mRNA destabilization [84], although depending on the context and complementarity, some miRNAs seem to preferentially act via translational repression [85]. miRNAs function in the context of a ribonucleoprotein complex called RNA-induced silencing complex (RISC), which includes the Argonaute (Ago) proteins. It is easy to imagine that of the approximately 2000 miRNAs that have so far been identified in the human genome, some of them could target similar sequences as RBPs and hinder RBP function (Figure 1C). Indeed, genome-wide analyses show that 3' UTR motifs that are recognized by both ARE-BPs and miRNAs are found on the same transcripts and may overlap [86,87].

As stated above, Hu proteins most often function as stabilizing factors, and there are several instances in the literature demonstrating competition between HuR and different miRNAs for control of gene expression. As a first example, HuR stabilizes the cationic acid transporter 1 (CAT1) mRNA and competes with miR-122 for control of its expression under stress-related conditions [88]. In Huh-7 hepatoma cells, HuR shuttles from the nucleus and relieves miR-122-mediated repression, resulting in the relocation of CAT1 mRNA from P-bodies to polysomes [88]. HuR also promotes the translation of topoisomerase IIα (TOP2A) mRNA and competes with miR-548c-3p for its expression [89]. In HeLa cells, inhibiting HuR or overexpressing miR-548c-3p alters TOP2A levels and controls the cellular response to the chemotherapeutic agent doxorubicin [89]. Also in HeLa cells, HuR stabilizes nucleolin (NCL) mRNA and relieves repression mediated by miR-494 [90]. In prostate cancer cells, HuR was shown to compete with miR-331-3p for regulation of erb-B2 receptor tyrosine kinase 2 (ERBB2) mRNA [91]. HuR also competes with miR-16, and in colorectal cancer cells, these two factors are antagonists in the regulation of cyclooxygenase-2 (COX2) mRNA [92]. HuR was shown to directly bind miR-16 *in vitro* and inhibit its activity in these transformed cells [92]. Conversely, miR-16 can prevent the HuR-mediated up-regulation of cyclin E1 in breast cancer cells [93].

In addition to the competition of miRNAs and ARE-BPs, there are a few examples in which these molecules cooperate to repress gene expression. One example is the ARE-targeting miR-16, which contains a sequence complementary to the ARE sequence. miR-16 promotes degradation of multiple mRNAs through its association with the ARE sequence, which requires a physical interaction between RISC and TTP, a destabilizing ARE-BP [94]. Another example of the cooperative activity between an RBP and a miRNA is HuR and let-7, in which HuR uncharacteristically recruits RISC/let-7 to an adjacent site on the c-myc mRNA, inhibiting its expression in HeLa cells [95]. Similarly, HuR and miR-19 cooperate to repress RhoB expression in HaCaT cells exposed to ultraviolet radiation [96]. One possible explanation for this unexpected effect of HuR is that binding of this RBP changes the conformation of the mRNA, helping unmask new miRNA binding sites [95,96]. A more detailed discussion of the above interactions can be found in the excellent review by Jiang and Coller [97]. Additionally, miRNAs could bring two non-contiguous sequences together creating new stem loops and RBP binding sites [98].

Since other Hu proteins have similar binding motifs to HuR, it is reasonable to think that these competitive and cooperative interactions may occur in neural cell types where HuR is less abundant but the other Hu proteins are highly expressed. Indeed, we showed that miR-129 can repress Kv1.1 mRNA in neurons and this repression can be reversed by HuD [60]. We previously identified three major HuD-binding motifs that are comprised of CU-rich (motif 1), GU-rich (motif 2), or AU-rich (motif 3) sequences [9]. Several miRNAs, such as miR-149-3p, miR-495-3p, and miR-590-3p, contain seed sequences that are complementary to these motifs, respectively, and could, therefore, be competitors of HuD under specific conditions. Finally, it is important to consider that miRNAs can directly affect the levels of RBPs such as in the case of miR-16 for HuR [99], miR-375 for HuD [100], and miR-206 for KSRP [101].

## 6. Conclusions

The competing RNA-protein and mRNA-miRNA networks outlined here have far reaching implications for regulation of gene expression in the nervous system. Several elements contribute to the competition within these networks, including temporal expression of the mRNA, miRNA, and RBPs, localized concentrations of these factors, binding/interaction site availabilities, and binding affinities. Thus, identifying miRNAs that could compete with RBPs may be informed by bioinformatics but cannot completely predict interactions that must take into account cell tropism, temporal expression, subcellular localization and other factors. With miRNAs and RBPs localizing into axons and dendrites [102,103,104], there is a high probability that these molecules compete for binding to targets in these processes, and new competitions could arise locally based on subcellular stoichiometry of different mRNAs, miRNAs, and RBPs. Undoubtedly, more genes are yet to be identified as shared targets of both miRNAs and RBPs, as well as shared targets of different RBPs. Uncovering the biological relevance of these targets will undoubtedly be facilitated by advances in bioinformatics, transcriptomics, and proteomics methodologies. However, heed must also be given to the physiological and cellular contexts of interactions, and unraveling these intricacies will likely require lower throughput cellular and molecular analyses. The highly regulated interactions between RBPs and their target sequences have been shown to contribute to the control of gene expression in a wide variety of physiological and pathological conditions from immune processes and cancer transformation [21] to muscle differentiation [26,27] and, importantly, to neural development and plasticity [105,106,107].
